# Salivary Exosomes as Nanocarriers for Cancer Biomarker Delivery

**DOI:** 10.3390/ma12040654

**Published:** 2019-02-21

**Authors:** Jordan Cheng, Taichiro Nonaka, David T.W. Wong

**Affiliations:** 1 Center for Oral/Head and Neck Oncology Research, School of Dentistry, University of California, Los Angeles, 10833 Le Conte Avenue, CHS 73-017, Los Angeles, CA 90095, USA; jcheng1@g.ucla.edu; 2 Division of Oral Biology and Medicine, School of Dentistry, University of California, Los Angeles, 10833 Le Conte Avenue, CHS 73-017, Los Angeles, CA 90095, USA

**Keywords:** salivary diagnostics, salivaomics, saliva-exosomics, biomarker, liquid biopsy, cancer, point-of-care

## Abstract

Human saliva is an ideal body fluid for developing non-invasive diagnostics. Saliva contains naturally-occurring nanoparticles with unique structural and biochemical characteristics. The salivary exosome, a nanoscale extracellular vesicle, has been identified as a highly informative nanovesicle with clinically-relevant information. Salivary exosomes have brought forth a pathway and mechanism by which cancer-derived biomarkers can be shuttled through the systemic circulation into the oral cavity. Despite such clinical potential, routine and reliable analyses of exosomes remain challenging due to their small sizes. Characterization of individual exosome nanostructures provides critical data for understanding their pathophysiological condition and diagnostic potential. In this review, we summarize a current array of discovered salivary biomarkers and nanostructural properties of salivary exosomes associated with specific cancers. In addition, we describe a novel electrochemical sensing technology, EFIRM (electric field-induced release and measurement), that advances saliva liquid biopsy, covering the current landscape of point-of-care saliva testing.

## 1. Introduction

In the era of personalized medicine, knowing specific cancer information is essential as it guides treatment decisions. Tissue biopsy is a standard method, but the limited sampling is often insufficient to capture the heterogeneity and evolution of tumors [1]. Conventional imaging techniques could offer non-invasive modalities, but they are less sensitive for early detection of cancer [2,3,4]. A developing concept, liquid biopsy, aims to provide an alternative to invasive tissue biopsy by identifying biomarkers in biofluids that reflect the presence of cancer [5]. Early pursuits in the liquid biopsy concept predominantly focused on blood, but now involve the analysis of urine, cerebral spinal fluid (CSF), and saliva. While blood, urine, and CSF are all viable candidates for cancer detection, saliva is the epitome of a non-invasive, readily-available, and easily-collectable biofluid. Saliva is composed of the secretions from the major salivary glands (parotid, submandibular, and sublingual) and numerous minor salivary glands [6]. Saliva has a wide variety of biological functions integral for food digestion and oral health maintenance [7]. Saliva contributes to the protection of teeth by pH maintenance and enamel remineralization [7]. Lactoferrin, lactoperoxidase, and immunoglobulin A contribute to saliva’s antibacterial and antiviral roles [8,9,10]. Since saliva reflects health conditions (e.g., blood glucose [11]) and provides unique information about the body (e.g., stress hormone [12]), rapid advances have been made in the field of salivary diagnostics.

Recently, extracellular vesicles (EVs) have gained considerable attention as mediators of intercellular signaling and as potential sources of cancer biomarkers [13]. Exosomes, which are nanoscale EVs of endocytic origin, were initially thought of as a way for cells to dispose of unnecessary proteins, but are now considered as mediators of intercellular signaling through RNA and functional protein exchange [14]. Exosomes are present in nearly all types of biofluids, carrying a tremendous potential for liquid biopsy and therapeutic applications [15]. There is a global demand for simple exosome isolation and robust analysis methods amenable to clinical application. Traditionally, exosomes are isolated through a density gradient or sucrose cushion by ultracentrifugation at 100,000× *g* [16]. Additional methods, however, such as polymer-assisted precipitation [17], immunoaffinity-based capture beads [18], immunoaffinity-based microfluidic chips [19], and acoustic fluidic chips [20], have surfaced with promising capabilities. Now, extracellular RNA (exRNA) can be screened in salivary exosomes in an attempt to detect and guide treatment for cancer [5].

Nanotechnology has become an important approach to improve the diagnosis and treatment of cancer [21]. Materials at the nanoscale have unique physical and biological properties that are useful for cancer detection [22]. Exosomes are naturally-occurring nanovesicles with clinically-relevant information and have the potential to reduce the detection limit of cancer biomarkers [23]. The concept that a patient can take a single drop of their own saliva and test it using a point-of-care device to determine their cancer risk has long been entertained by engineers and clinicians [24].

Here, we review the molecular and nanostructural properties of salivary exosomes as potential cancer biomarkers and discuss novel electrosensing technology that can detect and analyze salivary exosomes, with emphasis on point-of-care diagnosis.

## 2. Salivary Diagnostics

In the past decade, saliva researchers have explored saliva as a diagnostic fluid to detect oral and systemic diseases. Saliva is colorless, 99% water-based, slightly acidic (pH of 6.60), and contains a vast array of ions and organic compounds [25]. Salivary glands are densely surrounded by blood vessels containing epithelial cells enriched with passive and active cellular transporters and channels for substantial molecule exchange with circulating blood [26]. Proteomic studies of saliva revealed that 20–30% of the salivary proteome mirrors the plasma proteome, indicating that a substantial portion of salivary constituents are derived from the blood [27,28,29]. Thus, the significant overlap between saliva and blood due to their physiological interactions indicates a potential alternative approach to diagnosing systemic diseases. Saliva possesses several advantages over blood as a body fluid for clinical diagnosis. Saliva collection is performed easily and noninvasively, thereby reducing patient discomfort. Unlike blood, saliva does not coagulate, making it easier for handling and processing. Saliva is regarded as a mirror of oral and systemic health, containing a wide variety of biomarkers, rendering it an attractive biofluid for early disease detection. In fact, several studies have already demonstrated saliva’s usefulness for the diagnosis of health conditions such as diabetes [30], human immunodeficiency virus [31], cardiac disease [32], autoimmune diseases [33], and tobacco use [34]. Thus, many investigators have attempted to use saliva with point-of-care devices to assess health conditions (Table 1).

Salivaomics integrates the study of saliva and its constituents, functions, and related techniques [38,39]. Salivaomics technologies are derived from scientific advances in genomics, transcriptomics, and proteomics, and these high-throughput technologies have prompted interest in the use of saliva as a source of disease biomarkers. The development of particular saliva biomarkers and their associated in-clinic analyzers could facilitate point of care diagnostics [40].

The study of salivaomics is a relatively new field. It is only in the past decade that it has been known that salivary glands transfer molecular information. The full potential to harness this knowledge for biomedical use has been hampered due to difficulties in analyzing the heterogeneous nature of saliva. Saliva has been analyzed as a bulk population of constituents with insufficient sensitivity. Since the oral cavity is openly exposed to the surrounding environment, food and oral bacteria contribute to salivary composition [23]. The body’s natural circadian rhythm influences the production and composition of saliva [41]. A stimulated (masticatory) or rested salivary state also affects the saliva properties [42]. In addition, amylase, an example of an enzyme inherent in the saliva for the breakdown of complex carbohydrates, may interfere with or mask with diagnostic protein biomarkers during analysis [43]. Salivary proteins (histatins, statherin, or acidic proline-rich proteins) and RNAs are prone to degradation when taken out of their optimal environment [44]. Therefore, preemptive strategies must be used to stabilize the salivary components with protease inhibitors and RNase inhibitors to preserve their integrity [45,46]. In order to overcome these limitations, salivaomics should focus on salivary EVs. The EV fraction reduces the complexity of saliva, and the EV’s lipid bilayer protects its cargo, providing more stable and accurate clinically-relevant information for disease detection.

## 3. Salivary Exosomes

EVs are classified into three subgroups based on their size and associated pathways [47]. EVs include exosomes, microvesicles, and apoptotic bodies and can contain protein and genetic materials that resemble those in parental cells. Apoptotic bodies are generated in the terminal stages of apoptotic process and contain fragments of the nuclei and their cancerous mutations [48,49]. Among the different subpopulations of EVs, exosomes are of particular interest, as they are involved in cell-to-cell communication through RNA and protein exchange [15]. Exosomes are nano-sized vesicles (30–100 nm in diameter), originating from the endosomal pathway and secreted into the extracellular space by exocytosis. They are released from almost all cell types, transporting a unique cargo to the surrounding microenvironment and distal parts through vasculature. Exosomes have been isolated from a wide variety of body fluids such as blood, urine, saliva, breast milk, and cerebrospinal fluid [50,51]. Recent studies have shown that they play an important role in intercellular signaling and cellular homeostasis [52]. These functional roles are attributed to their contents originating from parental cells, thereby affecting the pathophysiological conditions of recipient cells. Given their biological role in cancer pathogenesis, exosomes can be used as ideal biomarkers in detecting and monitoring cancer.

The discovery that exosomes are present in saliva has raised a possible explanation for how cancer biomarkers are packaged and transported to the salivary glands [53]. The use of these small, but highly informative nanovesicles reduces the overall complexity of saliva [54]. The term “saliva-exosomics” is used to describe the study of salivary exosomes using “–omics” technologies (e.g., genomics, transcriptomics, or proteomics) and their relationship to biological functions in oral and systemic diseases [23]. Saliva-exosomics presents a new landscape and a new horizon of saliva biology that is just now being explored. 

### 3.1. Salivary Extracellular RNA 

The transcriptome is the complete set of RNA transcripts that are produced by the genome. It refers to all RNAs, including messenger RNA (mRNA), microRNA (miRNA), piwi-interacting RNA (piRNA), and other small RNAs such as rRNA and tRNA. The study of the salivary transcriptome uses high-throughput methods that have emerged as powerful tools for exploring biomarkers [55]. Saliva contains an assortment of extracellular RNA species including mRNA, miRNA, and other small non-coding RNAs (e.g., piRNA). The human salivary transcriptome was initially described using microarray technology [56]. This resulted in the characterizing of the salivary transcriptome as highly-fragmented coding and noncoding RNAs derived from host cells and oral microbiota [55,57,58]. High-throughput RNA sequencing (RNA-Seq) using human saliva revealed that the most abundant types of small RNAs are piRNA (7.5%) and miRNA (6.0%) [59]. Interestingly, miRNA [60,61] and piRNA [62,63] appear to be enriched at higher abundance in exosomes compared to whole saliva. This finding suggests that exosomes protect their cargo from degradation, making them attractive diagnostic tools for clinical application. miRNAs are a class of 21–25 nucleotide non-coding RNAs and of particularly interest since they play major roles in the regulation of gene expression in cancer cells [64]. In various cancers, such as oral, esophageal, lung, pancreatic, breast, and ovarian cancers, certain RNA biomarkers have been discovered in saliva and proposed as possible biomarkers (Table 2).

### 3.2. Salivary Circulating Tumor DNA 

Saliva contains cell-free DNA, and genomic analysis revealed that 70% is host-derived, whereas 30% originates from the oral microbiota [72]. Salivary DNA is stable and is of high quality, suggesting that salivary DNA is a useful biomarker target [73,74,75]. Circulating tumor DNA (ctDNA) is 180–200 base pair fragment of DNA containing mutated cancer sequences and believed to be derived from apoptotic or necrotic tumor cells releasing DNA fragments into circulation [76]. The DNA length, which is characteristic of the apoptotic process, corresponds to the inter-nucleosomal length of DNA that is wrapped around the nucleosome, including the linker segment [77].

There have been indications that ultrashort single-stranded cell-free DNA (<100 base pair) is present in plasma, which would suggest that these same ultrashort species could appear in saliva [78]. A large-scale study on multiple cancer types demonstrated that an increasing concentration of ctDNA is associated with an advancing stage of disease [79]. It is not clear if ctDNA has a pathophysiological role in promoting malignancy or is simply a waste-product of tumor cell death. There is some evidence, however, that the presence of ctDNA can promote cancer by transfecting healthy cells [80]. Given the heterogeneous nature of tumors, ctDNA analysis in liquid biopsy has the potential to detect accurately and monitor tumor progression in real time compared to tissue biopsy [5,77,81,82,83,84,85,86].

### 3.3. Salivary Protein Biomarkers

The discovery of salivary proteins associated with cancer has mainly been the result of high-throughput mass spectrometry screening of patient samples. From these studies, a series of protein biomarkers has been detected in whole saliva or salivary EVs for specific cancer types (Table 3). The emergence of the relevance of salivary EVs has provoked interest in EV-specific proteins, which have been found to be associated with oral and lung cancers. Although an extensive list of biomarkers has been compiled, further work must be performed to validate these candidate proteomic biomarkers in prospective clinical trials [87].

## 4. Nanostructural Properties of Salivary Exosomes

**Figure 1:**
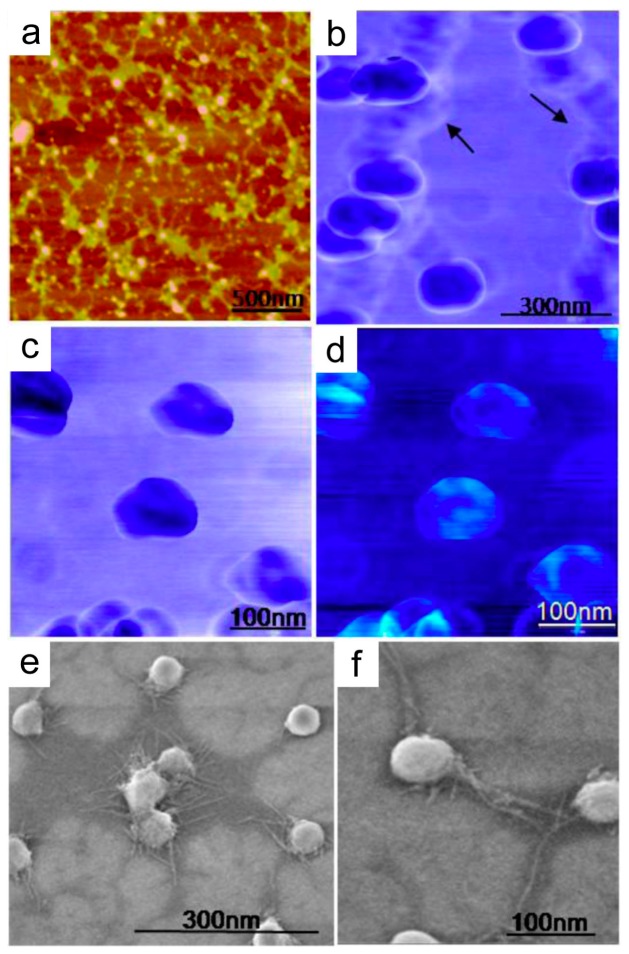
Nanostructure of individual salivary exosomes observed under tapping mode AFM and FESEM. (**a**) Tapping mode topographic low-force AFM image showing the round morphology of isolated exosomes. (**b**) AFM phase image of aggregated exosomes. Interconnections (arrows) lacking the characteristic phase shift probably indicate some extravesicular protein content. (**c**) At higher forces under AFM (~2 nN), representative single-exosome phase images reveal trilobed substructure within the center of the vesicles. The contrast in images may be presumably attributed to variable constitutive elements (lipid, protein, RNA ratio) making up these structures. (**d**) Corresponding height images show a central depression of the vesicles. (**e**) FESEM exosome image showing clumping exosomes and (**f**) single isolated vesicles as round bulging structures with well-resolved intervesicular connections. Reprinted with permission from [106]. Copyright 2010, American Chemical Society.

Salivary exosomes are naturally-occurring nanovesicles that are secreted from oral epithelial cells into saliva. Elucidating the nanostructural differences between the salivary exosomes originating from healthy subjects compared to patients with disease is particularly important as disease-specific exosomes may differ in functional properties [104]. In a nanostructural characterization study, the salivary exosomes from healthy donors using atomic force microscopy (AFM) and field emission scanning electron microscopy (FESEM) identified 70–100-nm exosomes with trilobed structures, demonstrating their reversible and elastic mechanical properties (Figure 1) [105,106]. Low-force imaging revealed round-shaped exosomes, suggesting exosomes have an inherent spherical morphology when stresses are not applied (Figure 1a,e,f). Additionally, AFM phase contrast images portrayed exosomes with a heterogeneous surface, likely attributed to the embedded proteins in a dense lipid membrane (Figure 1c,d).

**Figure 2:**
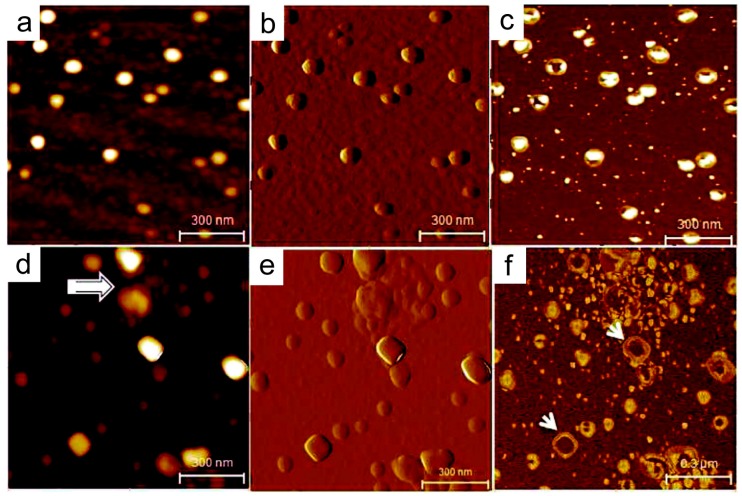
Structural characteristics of the human salivary exosome at the single-vesicle level. (**a**–**c**) AFM topographic (z = 0–10 nm), amplitude, and phase images of salivary exosomes from healthy donors. The exosomes appear as homogeneous circular structures with a distinct phase contrast between the less dense periphery and the denser core region. (**d**–**f**) Exosomes from an oral cancer patient show an irregular morphology with varying shapes and vesicle aggregation (arrows). (**e**) The amplitude image shows the clumping of vesicles. (**f**) In the phase image, the larger vesicles appear hollow (arrows) without the dense core region typically seen in normal exosomes. All images were obtained over mica substrates under ambient conditions. Reprinted with permission from [107]. Copyright 2011, American Chemical Society.

**Figure 3:**
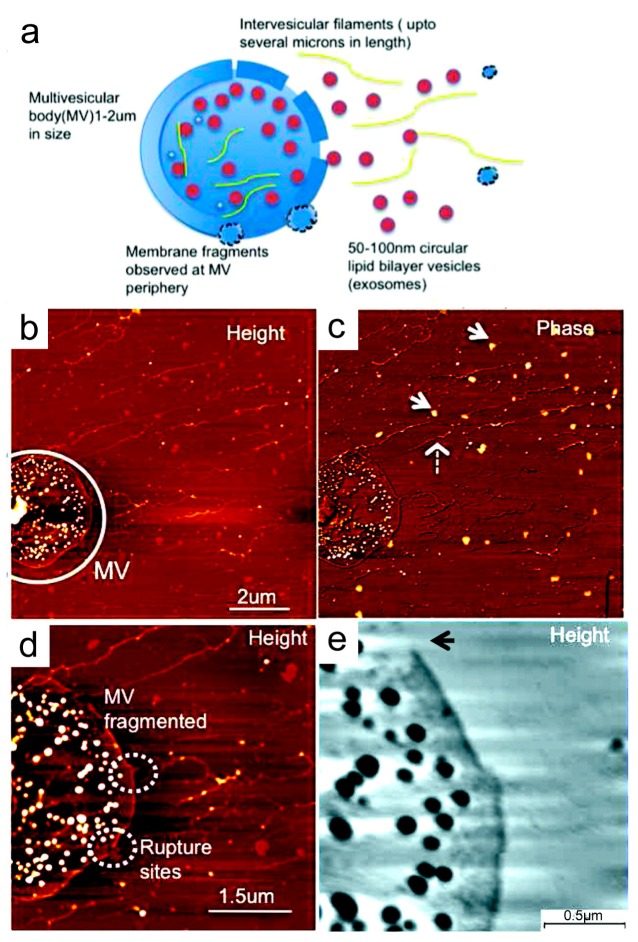
Release of exosomes from multivesicular bodies (MVs) seen in the saliva of an oral cancer patient. (**a**) Schematic of single MV membrane rupture and exosome release along with intervesicular filaments from MV lumen. (**b**) AFM topographic and phase image of a single MV filled with exosome vesicles. (**c**) Elongated intervesicular filaments (dashed arrow) and exosome-like vesicles (arrows) are observed. (**d**) At high resolution, the ruptures and fragmentation of the MV membrane are clearly observed (dashed circles). Additionally, the intervesicular filaments are seen in the MV lumen. (**e**) At higher resolution, a large rupture is seen in the MV membrane (arrow). Samples were imaged under ambient conditions. Reprinted with permission from [107]. Copyright 2011, American Chemical Society.

Morphological characterization of salivary exosomes at the single-vesicle level using high-resolution AFM displayed irregular morphologies and higher intervesicular aggregation in an oral cancer patient compared to a healthy control (Figure 2) [107]. Quantitative analysis also revealed that size and CD63 surface density were significantly increased in cancer exosomes (98.3 ± 4.6 nm) compared to normal exosomes (67.4 ± 2.9 nm) (*p* < 0.05) [107]. Structural and morphological aberrations in the exosomes are suggestive that these exosomes are at least in part cancer-derived products that were shed directly into saliva. Multivesicular bodies (MVs) were identified in oral cancer salivary exosome fractions (Figure 3). These multivesicular structures showcased ruptures and elongated nanofilaments around the lumen of these MVs, suggesting that these are the sites for exosome release, as well as filamentous extension of nucleic acids. These images suggest that oral cancer-derived exosomes in saliva have distinct properties that make them potential biomarkers for cancer diagnosis. 

## 5. Electric Field-Induced Release and Measurement 

Routine isolation and analysis of nanoscale exosomes in clinical settings is challenging. Conventional methods have been facing a number of drawbacks including high cost and long processing time. The current gold standard for exosome isolation is by density gradient or sucrose cushion ultracentrifugation at 100,000 × *g* [16]. Regular laboratory methods for the ctDNA interrogation include allele-specific polymerase chain reaction (PCR), digital PCR, and next-generation sequencing (NGS) [108]. These methods are predominantly PCR-based and have demonstrated limited success in saliva. In one study, the saliva from 93 head and neck squamous cell carcinoma (HNSCC) patients were analyzed for human papilloma virus (HPV) DNA (HPV16/18) and/or somatic mutations (*TP53, PIK3CA, CDKN2A, FBXW7, HRAS,* and *NRAS*) related to HNSCC using a multiplex panel of PCR primers [109]. Their results showed that in saliva from oral cavity cancers, ctDNAs associated with HNSCC were detected in saliva with 100% concordance. However, saliva from patients from other anatomical sites demonstrated poor results with oropharynx (47%), larynx (70%), and hypopharynx (67%). Thus, the development of reliable and highly-sensitive modalities for cancer detection is an unmet need [110,111,112].

**Figure 4:**
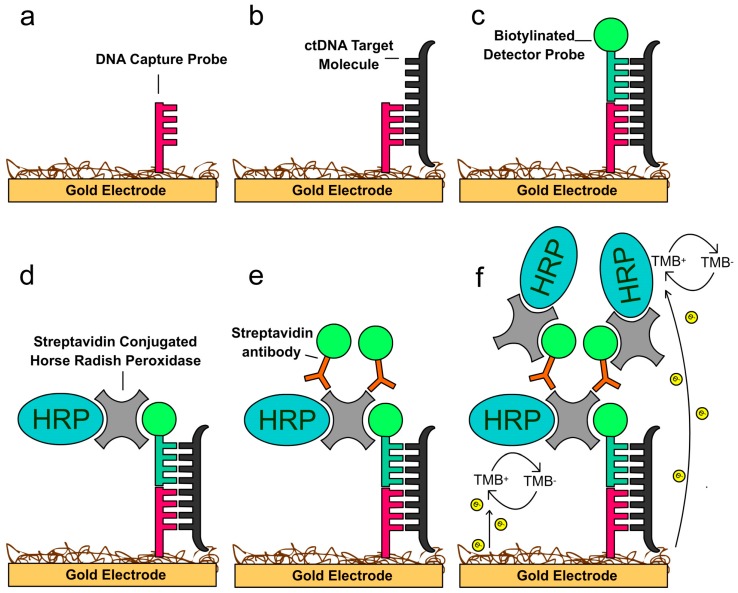
Schematic of the EFIRM assay procedure. (**a**) An electrical field is applied to polymerize pyrrole in order to anchor a single-stranded oligonucleotide capture probe specific for a ctDNA onto a gold electrode. (**b**) The saliva containing ctDNA target molecules is added and hybridizes with the capture probe in the presence of a cyclical square wave. (**c**) A complementary biotinylated single-stranded oligonucleotide detector probe hybridizes with the target under an electric field. (**d**) HRP (horseradish peroxidase)-streptavidin binds to biotin on the detector probe. (**e**,**f**) A subsequent layer of biotinylated anti-streptavidin antibody and HRP-streptavidin amplifies the signal. The 3,3’,5,5’-tetramethylbenzidine (TMB) substrate is added to generate a continuous, quantifiable electric current through a reduction reaction with HRP.

**Figure 5:**
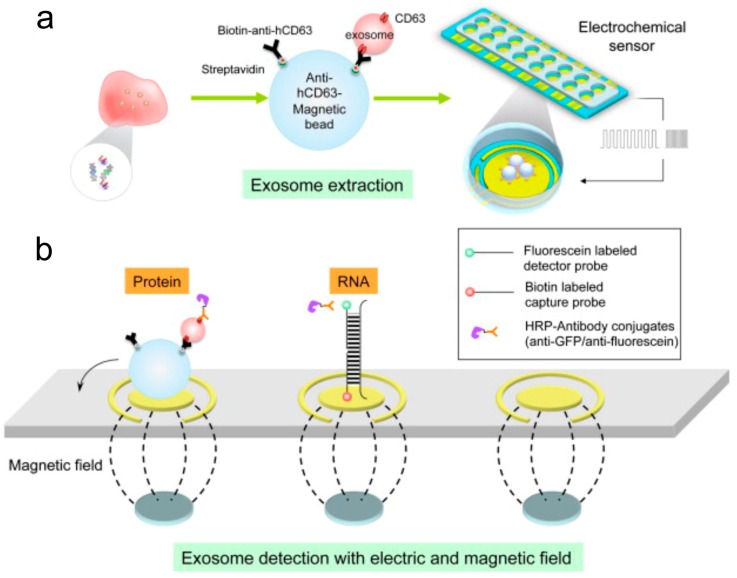
Schematic of the integrated magnetic-electrochemical electric field-induced release and measurement (EFIRM). (**a**) Magnetic beads conjugated with an exosome surface marker CD63 antibody capture salivary exosome. (**b**) Magnetic field capture of exosomes and electric field release of RNA.

**Figure 6:**
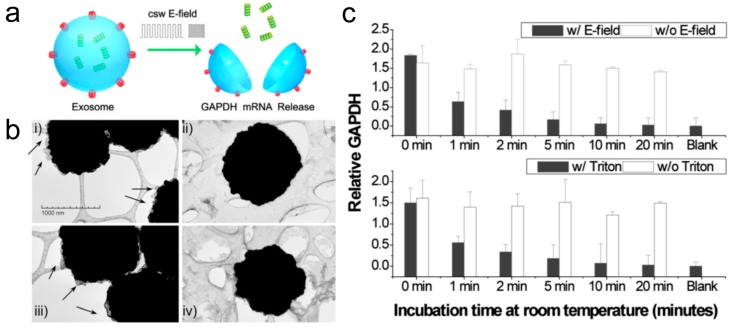
EFIRM can disrupt exosomes to release exosomal GAPDH mRNA from human saliva. (**a**) Schematic illustration of an exosome disrupted with an electric field (E-field) and GADPH mRNA released. (**b**) Transmission electron microscopy (TEM) images before (i and iii) and after (ii and iv); E-field (top) or Triton X-100 detergent (bottom) treatment. (i and iii) show exosomes (arrows) attached to anti-CD63 antibody-conjugated magnetic beads; (ii and iv) indicate the absence of exosomes disrupted after treatment with (ii) a CSW E-field for 200 s or (iv) with Triton X-100 for 20 min. Background webbing indicates lacey support film for TEM. (**c**) The levels of GAPDH mRNAs were measured at different time points by EFIRM after the application of E-field or Triton X-100. The kinetics of the GAPDH mRNA signal reduction by EFIRM (top) or Triton X-100 (bottom) indicates that bare RNAs (released by E-field or Triton X-100) decay rapidly.

Recently, dramatic progress has been made in nanotechnology by bringing new electrochemical biosensing technology to exosome analysis. The electrochemical sensing approach is highly suitable for the detection of biomolecules due to its inherent advantages such as high sensitivity and specificity [113,114,115]. In comparison with conventional methods (e.g. PCR, NGS), electrochemical techniques are fast and affordable, only requiring small sample volumes less than 50 μL. We have developed a novel saliva liquid biopsy technology termed EFIRM (electric field-induced release and measurement), which has been engineered to detect minute amounts of ctDNA and RNA in saliva (Figure 4) [116]. This procedure can detect and quantify the ctDNA in 40 μL of saliva of non-small cell lung cancer (NSCLC) patients with near perfect concordance with biopsy genotyping (96–100%) [116,117]. This non-PCR-based electrochemical platform utilizes an immobilized oligonucleotide capture probe and detector probe system aided by cyclic square wave (CSW) voltammetry throughout the procedure. Moreover, the EFIRM platform can integrate magnetic selection and electrochemical detection, thereby facilitating the capturing of exosomes present in saliva (Figure 5) [118]. For detection, exosomes are first captured onto magnetic beads conjugated with antibody against CD63, a representative exosome surface marker. The CSW electric field is then applied to release the RNA from the exosomes, followed by a mixture of detector probes. The HRP-conjugated secondary antibody and 3,3′,5,5′-tetramethylbenzidine (TMB) substrate generate electrical current and detected by an electric sensor. Indeed, this integrated magnetic-electrochemical EFIRM can detect and analyze exosomes by disrupting exosomes and releasing GAPDH mRNA in a similar manner as Triton X-100 detergent lysis (Figure 6) [118]. Combining affinity capture and electrochemical sensing technologies is desirable for rapid detection of exosomal biomarkers, facilitating the development of point-of-care devices and their translation into routine clinical use.

## 6. Conclusions and Future Perspectives

Nanodiagnostic strategies are being developed to meet the requirements of clinical practice for the detection of cancer. Nanovesicles and their detection method play vital roles in the development of new platforms for biomarker detection essential for diagnosis and treatment decisions. The versatile structural and functional properties of exosomes pave the way for the development of specific and sensitive diagnostics, opening the door for precise personalized medicine. Further nanostructural and functional studies of salivary exosomes are warranted for a better understanding of the biological mechanisms mediated by molecules present in exosomes.

Many transcriptomic and proteomic studies have uncovered potential salivary biomarkers, placing greater focus on exosomes and their disease-associated biomarkers. EFIRM identification of actionable *EGFR* ctDNA (L858R and Exon19 deletion) in saliva will determine which tyrosine kinase inhibitors (TKIs) are to be prescribed [116]. If ctDNA can be frequently and routinely assayed in saliva, the early detection of mutation T790M, another *EGFR* mutation indicative of resistance to first and second-generation TKIs, would influence changes in therapy [119]. Despite such clinical potential of saliva, reliable exosome analyses remain challenging due to their small sizes [120]. The major technical challenge in exosome detection in clinical applications is to detect disease-specific exosomes in heterogeneous bulk populations derived from normal and cancer cells. The electrochemical sensing approach with rapid and sensitive readout is an effective detection modality. The integration of magnetic-electrochemical approaches together with other novel platforms (e.g., microfluidics) can result in an efficient tool for clinical diagnosis, particularly in point-of-care devices for a wide range of disease detection. 

The development of multiplex detection technologies (e.g., nano flow cytometry) will offer insight into understanding the exosome’s heterogeneity and subset differentiation. Combining the outstanding components of exosome isolation techniques and multiplexed assay systems will also make it capable of selective isolation of specific exosome subtypes in heterogeneous samples. This can expand the borders of saliva research and open up new avenues of biomarker discovery and therapeutic interventions. As research and knowledge in the field of salivaomics and saliva-exosomics continues to advance, it will solidify saliva as an integral part of liquid biopsy. When these conditions are met, saliva liquid biopsy will be a viable tool for high-risk population screening.

## Figures and Tables

**Table 1:** Salivary diagnosis for health conditions.

Disease/Condition	Platform	Salivary Biomarker	Reference
Diabetes	Screen-printed electro chemical sensor	Glucose	[30]
HIV	OraQuick	HIV-1/2 antibody	[31]
Hepatitis C	Mono-Lisa anti-HCV Plus	HCV antibody	[35]
Acute myocardial infarction	Luminex lab-on-a-chip	C-reactive protein, myoglobin, MMP-9, IL-1B, slCAM-1, myeloperoxidase	[32]
Asthma and chronic obstructive pulmonary disease (COPD)	Multiplexed fiber optic microsphere-based cytokine array	IFNg, IP-10, RANTES, eotaxin-3, VEGF	[36]
Periodontitis	Lab-on-a-chip	C-reactive protein, MMP-8, IL-1B	[37]
Tobacco use	NicAlert test strips	Cotinine	[34]

**Table 2:** Salivary RNA biomarkers in cancers.

Cancer	RNA Type	Salivary RNA Biomarker	Reference
Oral cancer	messenger RNA	*DUSP1*, *H3F3A*, *IL1B*, *IL8*, *OAZ1*, *S100P*, *SAT*	[65]
	microRNA	miR-125a, miR-200a	[66]
Esophageal cancer	microRNA	miR-144, miR-451, miR-98, miR-10b, miR-363	[67]
Lung cancer	messenger RNA	*CCNI*, *FGF19*, *GREB1*, *FRS2*, *EGFR*	[68]
Pancreatic cancer	messenger RNA	*KRAS*, *MBD3L2*, *ACRV1*, *DPM1*	[69]
Breast cancer	messenger RNA	*CSTA*, *TPT1*, *IGF2BP1*, *GRM1*, *GRIK1*, *H6PD*, *MDM4*, *S100A8*	[70]
Ovarian cancer	messenger RNA	*AGPAT1*, *B2M*, *IER3*, *IL1B*, *BASP1*	[71]

**Table 3:** Salivary protein biomarkers for cancers.

Cancer	Sample	Salivary Protein Biomarker	Reference
Breast cancer	Whole saliva	EGF	[88]
		c-erbB-2	[89]
		CA15-3, c-erbB-2	[90]
		VEGF, EGF, CEA	[91]
		CA6	[70]
		LRP	[92]
Oral cancer	Whole saliva	A1BG, CFB	[93]
		M2BP, MRP14, CD59, CAT, PFN	[94]
		FGB, S100, TF, IGHG, CFL1	[95]
		ADA	[96]
		IL-8, M2BP, IL-1B	[97]
	Salivary EVs	A2M, HPa, MUC5B, LGALS3BP, IGHA1, PIP, PKM1/M2, GAPDH	[98]
Gastric cancer	Whole saliva	1472.78Da, 2936.49Da, 6556.81Da, 7081.17Da	[99]
		CSTB, TPI1, DMBT1, CALML3, IGH, IL1RA	[100]
Lung cancer	Whole saliva	HP, AZGP1, CALPR	[101]
	Salivary EVs	Annexin A1, A2, A3, A5, A6, A11, NPRL2, CEACAM1, HIST1H4A, MUC1, PROM1, TNFAIP3	[102]
Ovarian cancer	Whole saliva	CA125	[103]

## References

[1] Gerlinger M., Rowan A.J., Horswell S., Math M., Larkin J., Endesfelder D., Gronroos E., Martinez P., Matthews N., Stewart A. (2012). Intratumor heterogeneity and branched evolution revealed by multiregion sequencing. N. Engl. J. Med..

[2] Brindle K. (2008). New approaches for imaging tumour responses to treatment. Nat. Rev. Cancer.

[3] Condeelis J., Weissleder R. (2010). In vivo imaging in cancer. Cold Spring Harb. Perspect. Biol..

[4] Weissleder R., Pittet M.J. (2008). Imaging in the era of molecular oncology. Nature.

[5] Nonaka T., Wong D.T.W. (2018). Liquid Biopsy in Head and Neck Cancer: Promises and Challenges. J. Dent. Res..

[6] Lamy E., Mau M. (2012). Saliva proteomics as an emerging, non-invasive tool to study livestock physiology, nutrition and diseases. J. Proteomics.

[7] Dawes C., Pedersen A.M., Villa A., Ekstrom J., Proctor G.B., Vissink A., Aframian D., McGowan R., Aliko A., Narayana N. (2015). The functions of human saliva: A review sponsored by the World Workshop on Oral Medicine VI. Arch. Oral Biol..

[8] Humphrey S.P., Williamson R.T. (2001). A review of saliva: Normal composition, flow, and function. J. Prosthet. Dent..

[9] Miller C.S., King C.P., Langub M.C., Kryscio R.J., Thomas M.V. (2006). Salivary biomarkers of existing periodontal disease: A cross-sectional study. J. Am. Dent. Assoc..

[10] Schipper R.G., Silletti E., Vingerhoeds M.H. (2007). Saliva as research material: biochemical, physicochemical and practical aspects. Arch. Oral Biol..

[11] Gupta S., Sandhu S.V., Bansal H., Sharma D. (2015). Comparison of salivary and serum glucose levels in diabetic patients. J. Diabetes Sci. Technol..

[12] Raff H., Findling J.W. (2010). Biomarkers: Salivary cortisol or cortisone?. Nat. Rev. Endocrinol..

[13] Becker A., Thakur B.K., Weiss J.M., Kim H.S., Peinado H., Lyden D. (2016). Extracellular Vesicles in Cancer: Cell-to-Cell Mediators of Metastasis. Cancer Cell.

[14] Mathieu M., Martin-Jaular L., Lavieu G., Thery C. (2019). Specificities of secretion and uptake of exosomes and other extracellular vesicles for cell-to-cell communication. Nat. Cell Biol..

[15] Simons M., Raposo G. (2009). Exosomes-vesicular carriers for intercellular communication. Curr. Opin. Cell Biol..

[16] Thery C., Amigorena S., Raposo G., Clayton A. (2006). Isolation and characterization of exosomes from cell culture supernatants and biological fluids. Curr. Protoc. Cell Biol..

[17] Niu Z., Pang R.T.K., Liu W., Li Q., Cheng R., Yeung W.S.B. (2017). Polymer-based precipitation preserves biological activities of extracellular vesicles from an endometrial cell line. PLoS ONE.

[18] Sharma P., Ludwig S., Muller L., Hong C.S., Kirkwood J.M., Ferrone S., Whiteside T.L. (2018). Immunoaffinity-based isolation of melanoma cell-derived exosomes from plasma of patients with melanoma. J. Extracell. Vesicles.

[19] He M., Zeng Y. (2016). Microfluidic Exosome Analysis toward Liquid Biopsy for Cancer. J. Lab. Autom..

[20] Wu M., Ouyang Y., Wang Z., Zhang R., Huang P.H., Chen C., Li H., Li P., Quinn D., Dao M. (2017). Isolation of exosomes from whole blood by integrating acoustics and microfluidics. Proc. Natl. Acad. Sci. USA.

[21] Morris S.A., Farrell D., Grodzinski P. (2014). Nanotechnologies in cancer treatment and diagnosis. J. Natl. Compr. Cancer Netw..

[22] Choi Y.E., Kwak J.W., Park J.W. (2010). Nanotechnology for early cancer detection. Sensors.

[23] Nonaka T., Wong D.T.W. (2017). Saliva-Exosomics in Cancer: Molecular Characterization of Cancer-Derived Exosomes in Saliva. Enzymes.

[24] Aro K., Wei F., Wong D.T., Tu M. (2017). Saliva Liquid Biopsy for Point-of-Care Applications. Front Public Health.

[25] Carpenter G.H. (2013). The secretion, components, and properties of saliva. Annu. Rev. Food Sci. Technol..

[26] Groschl M. (2008). Current status of salivary hormone analysis. Clin. Chem..

[27] Yeh C.K., Christodoulides N.J., Floriano P.N., Miller C.S., Ebersole J.L., Weigum S.E., McDevitt J., Redding S.W. (2010). Current development of saliva/oral fluid-based diagnostics. Tex Dent. J..

[28] Bandhakavi S., Stone M.D., Onsongo G., Van Riper S.K., Griffin T.J. (2009). A dynamic range compression and three-dimensional peptide fractionation analysis platform expands proteome coverage and the diagnostic potential of whole saliva. J. Proteome Res..

[29] Yan W., Apweiler R., Balgley B.M., Boontheung P., Bundy J.L., Cargile B.J., Cole S., Fang X., Gonzalez-Begne M., Griffin T.J. (2009). Systematic comparison of the human saliva and plasma proteomes. Proteomics Clin. Appl..

[30] Du Y., Zhang W., Wang M.L. (2016). Sensing of Salivary Glucose Using Nano-Structured Biosensors. Biosensors.

[31] Zachary D., Mwenge L., Muyoyeta M., Shanaube K., Schaap A., Bond V., Kosloff B., de Haas P., Ayles H. (2012). Field comparison of OraQuick ADVANCE Rapid HIV-1/2 antibody test and two blood-based rapid HIV antibody tests in Zambia. BMC Infect. Dis..

[32] Floriano P.N., Christodoulides N., Miller C.S., Ebersole J.L., Spertus J., Rose B.G., Kinane D.F., Novak M.J., Steinhubl S., Acosta S. (2009). Use of saliva-based nano-biochip tests for acute myocardial infarction at the point of care: a feasibility study. Clin. Chem..

[33] Lee Y.H., Wong D.T. (2009). Saliva: An emerging biofluid for early detection of diseases. Am. J. Dent..

[34] Cooke F., Bullen C., Whittaker R., McRobbie H., Chen M.H., Walker N. (2008). Diagnostic accuracy of NicAlert cotinine test strips in saliva for verifying smoking status. Nicot. Tob. Res..

[35] Van Doornum G.J., Lodder A., Buimer M., van Ameijden E.J., Bruisten S. (2001). Evaluation of hepatitis C antibody testing in saliva specimens collected by two different systems in comparison with HCV antibody and HCV RNA in serum. J. Med. Virol..

[36] Walt D.R., Blicharz T.M., Hayman R.B., Rissin D.M., Bowden M., Siqueira W.L., Helmerhorst E.J., Grand-Pierre N., Oppenheim F.G., Bhatia J.S. (2007). Microsensor arrays for saliva diagnostics. Ann. N. Y. Acad. Sci..

[37] Christodoulides N., Floriano P.N., Miller C.S., Ebersole J.L., Mohanty S., Dharshan P., Griffin M., Lennart A., Ballard K.L., King C.P. (2007). Lab-on-a-chip methods for point-of-care measurements of salivary biomarkers of periodontitis. Ann. N. Y. Acad. Sci..

[38] Ai J., Smith B., Wong D.T. (2010). Saliva ontology: an ontology-based framework for a Salivaomics Knowledge Base. BMC Bioinf..

[39] Wong D.T. (2012). Salivaomics. J. Am. Dent. Assoc..

[40] Herr A.E., Hatch A.V., Throckmorton D.J., Tran H.M., Brennan J.S., Giannobile W.V., Singh A.K. (2007). Microfluidic immunoassays as rapid saliva-based clinical diagnostics. Proc. Natl. Acad. Sci. USA.

[41] Ferguson D.B., Botchway C.A. (1979). Circadian variations in the flow rate and composition of whole saliva stimulated by mastication. Arch. Oral Biol..

[42] Mackie D.A., Pangborn R.M. (1990). Mastication and its influence on human salivary flow and alpha-amylase secretion. Physiol. Behav..

[43] Henson B.S., Wong D.T. (2010). Collection, storage, and processing of saliva samples for downstream molecular applications. Methods Mol. Biol..

[44] Helmerhorst E.J., Oppenheim F.G. (2007). Saliva: A dynamic proteome. J. Dent. Res..

[45] Chiang S.H., Thomas G.A., Liao W., Grogan T., Buck R.L., Fuentes L., Yakob M., Laughlin M.J., Schafer C., Nazmul-Hossain A. (2015). RNAPro*SAL: a device for rapid and standardized collection of saliva RNA and proteins. Biotechniques.

[46] Xiao H., Wong D.T. (2012). Method development for proteome stabilization in human saliva. Anal. Chim. Acta..

[47] Kalra H., Simpson R.J., Ji H., Aikawa E., Altevogt P., Askenase P., Bond V.C., Borras F.E., Breakefield X., Budnik V. (2012). Vesiclepedia: A compendium for extracellular vesicles with continuous community annotation. PLoS Biol..

[48] Samos J., Garcia-Olmo D.C., Picazo M.G., Rubio-Vitaller A., Garcia-Olmo D. (2006). Circulating nucleic acids in plasma/serum and tumor progression: Are apoptotic bodies involved? An experimental study in a rat cancer model. Ann. N. Y. Acad. Sci..

[49] Taylor R.C., Cullen S.P., Martin S.J. (2008). Apoptosis: Controlled demolition at the cellular level. Nat. Rev. Mol. Cell Biol..

[50] Andaloussi S.E., Mager I., Breakefield X.O., Wood M.J. (2013). Extracellular vesicles: biology and emerging therapeutic opportunities. Nat. Rev. Drug Discov..

[51] Lasser C., Alikhani V.S., Ekstrom K., Eldh M., Paredes P.T., Bossios A., Sjostrand M., Gabrielsson S., Lotvall J., Valadi H. (2011). Human saliva, plasma and breast milk exosomes contain RNA: uptake by macrophages. J. Transl. Med..

[52] Takahashi A., Okada R., Nagao K., Kawamata Y., Hanyu A., Yoshimoto S., Takasugi M., Watanabe S., Kanemaki M.T., Obuse C. (2017). Exosomes maintain cellular homeostasis by excreting harmful DNA from cells. Nat. Commun..

[53] Ogawa Y., Kanai-Azuma M., Akimoto Y., Kawakami H., Yanoshita R. (2008). Exosome-like vesicles with dipeptidyl peptidase IV in human saliva. Biol. Pharm. Bull..

[54] Al-Tarawneh S.K., Border M.B., Dibble C.F., Bencharit S. (2011). Defining salivary biomarkers using mass spectrometry-based proteomics: A systematic review. OMICS.

[55] Park N.J., Li Y., Yu T., Brinkman B.M., Wong D.T. (2006). Characterization of RNA in saliva. Clin. Chem..

[56] Li Y., Zhou X., St John M.A., Wong D.T. (2004). RNA profiling of cell-free saliva using microarray technology. J. Dent. Res..

[57] Park N.J., Zhou X., Yu T., Brinkman B.M., Zimmermann B.G., Palanisamy V., Wong D.T. (2007). Characterization of salivary RNA by cDNA library analysis. Arch. Oral Biol..

[58] Spielmann N., Ilsley D., Gu J., Lea K., Brockman J., Heater S., Setterquist R., Wong D.T. (2012). The human salivary RNA transcriptome revealed by massively parallel sequencing. Clin. Chem..

[59] Bahn J.H., Zhang Q., Li F., Chan T.M., Lin X., Kim Y., Wong D.T., Xiao X. (2015). The landscape of microRNA, Piwi-interacting RNA, and circular RNA in human saliva. Clin. Chem..

[60] Gallo A., Tandon M., Alevizos I., Illei G.G. (2012). The majority of microRNAs detectable in serum and saliva is concentrated in exosomes. PLoS ONE.

[61] Michael A., Bajracharya S.D., Yuen P.S., Zhou H., Star R.A., Illei G.G., Alevizos I. (2010). Exosomes from human saliva as a source of microRNA biomarkers. Oral. Dis..

[62] Ogawa Y., Taketomi Y., Murakami M., Tsujimoto M., Yanoshita R. (2013). Small RNA transcriptomes of two types of exosomes in human whole saliva determined by next generation sequencing. Biol. Pharm. Bull..

[63] Ogawa Y., Tsujimoto M., Yanoshita R. (2016). Next-Generation sequencing of protein-coding and long non-protein-coding RNAs in two types of exosomes derived from Human whole saliva. Biol. Pharm. Bull..

[64] Ha M., Kim V.N. (2014). Regulation of microRNA biogenesis. Nat. Rev. Mol. Cell Biol..

[65] Li Y., St John M.A., Zhou X., Kim Y., Sinha U., Jordan R.C., Eisele D., Abemayor E., Elashoff D., Park N.H. (2004). Salivary transcriptome diagnostics for oral cancer detection. Clin. Cancer Res..

[66] Matse J.H., Yoshizawa J., Wang X., Elashoff D., Bolscher J.G., Veerman E.C., Bloemena E., Wong D.T. (2013). Discovery and prevalidation of salivary extracellular microRNA biomarkers panel for the noninvasive detection of benign and malignant parotid gland tumors. Clin. Cancer Res..

[67] Du J., Zhang L. (2017). Analysis of salivary microRNA expression profiles and identification of novel biomarkers in esophageal cancer. Oncol. Lett..

[68] Zhang L., Xiao H., Zhou H., Santiago S., Lee J.M., Garon E.B., Yang J., Brinkmann O., Yan X., Akin D. (2012). Development of transcriptomic biomarker signature in human saliva to detect lung cancer. Cell Mol. Life Sci..

[69] Zhang L., Farrell J.J., Zhou H., Elashoff D., Akin D., Park N.H., Chia D., Wong D.T. (2010). Salivary transcriptomic biomarkers for detection of resectable pancreatic cancer. Gastroenterology.

[70] Zhang L., Xiao H., Karlan S., Zhou H., Gross J., Elashoff D., Akin D., Yan X., Chia D., Karlan B. (2010). Discovery and preclinical validation of salivary transcriptomic and proteomic biomarkers for the non-invasive detection of breast cancer. PLoS ONE.

[71] Lee Y.H., Kim J.H., Zhou H., Kim B.W., Wong D.T. (2012). Salivary transcriptomic biomarkers for detection of ovarian cancer: For serous papillary adenocarcinoma. J. Mol. Med. (Berl).

[72] Rylander-Rudqvist T., Hakansson N., Tybring G., Wolk A. (2006). Quality and quantity of saliva DNA obtained from the self-administrated oragene method-a pilot study on the cohort of Swedish men. Cancer Epidemiol. Biomarkers Prev..

[73] Bonne N.J., Wong D.T. (2012). Salivary biomarker development using genomic, proteomic and metabolomic approaches. Genome Med..

[74] Hansen T.V., Simonsen M.K., Nielsen F.C., Hundrup Y.A. (2007). Collection of blood, saliva, and buccal cell samples in a pilot study on the Danish nurse cohort: comparison of the response rate and quality of genomic DNA. Cancer Epidemiol. Biomarkers Prev..

[75] Looi M.L., Zakaria H., Osman J., Jamal R. (2012). Quantity and quality assessment of DNA extracted from saliva and blood. Clin. Lab..

[76] Jahr S., Hentze H., Englisch S., Hardt D., Fackelmayer F.O., Hesch R.D., Knippers R. (2001). DNA fragments in the blood plasma of cancer patients: Quantitations and evidence for their origin from apoptotic and necrotic cells. Cancer Res..

[77] Diaz L.A., Bardelli A. (2014). Liquid biopsies: genotyping circulating tumor DNA. J. Clin. Oncol..

[78] Burnham P., Kim M.S., Agbor-Enoh S., Luikart H., Valantine H.A., Khush K.K., De Vlaminck I. (2016). Single-stranded DNA library preparation uncovers the origin and diversity of ultrashort cell-free DNA in plasma. Sci. Rep..

[79] Bettegowda C., Sausen M., Leary R.J., Kinde I., Wang Y., Agrawal N., Bartlett B.R., Wang H., Luber B., Alani R.M. (2014). Detection of circulating tumor DNA in early- and late-stage human malignancies. Sci. Transl. Med..

[80] Garcia-Olmo D.C., Dominguez C., Garcia-Arranz M., Anker P., Stroun M., Garcia-Verdugo J.M., Garcia-Olmo D. (2010). Cell-free nucleic acids circulating in the plasma of colorectal cancer patients induce the oncogenic transformation of susceptible cultured cells. Cancer Res..

[81] Aarthy R., Mani S., Velusami S., Sundarsingh S., Rajkumar T. (2015). Role of Circulating Cell-Free DNA in Cancers. Mol. Diagn. Ther..

[82] Chaudhuri A.A., Binkley M.S., Osmundson E.C., Alizadeh A.A., Diehn M. (2015). Predicting Radiotherapy Responses and Treatment Outcomes Through Analysis of Circulating Tumor DNA. Semin. Radiat. Oncol..

[83] Ichihara E., Lovly C.M. (2015). Shades of T790M: Intratumor Heterogeneity in EGFR-Mutant Lung Cancer. Cancer Discov..

[84] Ignatiadis M., Lee M., Jeffrey S.S. (2015). Circulating Tumor Cells and Circulating Tumor DNA: Challenges and Opportunities on the Path to Clinical Utility. Clin. Cancer Res..

[85] Piotrowska Z., Niederst M.J., Karlovich C.A., Wakelee H.A., Neal J.W., Mino-Kenudson M., Fulton L., Hata A.N., Lockerman E.L., Kalsy A. (2015). Heterogeneity Underlies the Emergence of EGFRT790 Wild-Type Clones Following Treatment of T790M-Positive Cancers with a Third-Generation EGFR Inhibitor. Cancer Discov..

[86] Polivka J., Pesta M., Janku F. (2015). Testing for oncogenic molecular aberrations in cell-free DNA-based liquid biopsies in the clinic: are we there yet?. Expert Rev. Mol. Diagn..

[87] Pepe M.S., Etzioni R., Feng Z., Potter J.D., Thompson M.L., Thornquist M., Winget M., Yasui Y. (2001). Phases of biomarker development for early detection of cancer. J. Natl. Cancer Inst..

[88] Navarro M.A., Mesia R., Diez-Gibert O., Rueda A., Ojeda B., Alonso M.C. (1997). Epidermal growth factor in plasma and saliva of patients with active breast cancer and breast cancer patients in follow-up compared with healthy women. Breast Cancer Res. Treat..

[89] Streckfus C., Bigler L., Dellinger T., Dai X., Kingman A., Thigpen J.T. (2000). The presence of soluble c-erbB-2 in saliva and serum among women with breast carcinoma: a preliminary study. Clin. Cancer Res..

[90] Streckfus C., Bigler L., Tucci M., Thigpen J.T. (2000). A preliminary study of CA15-3, c-erbB-2, epidermal growth factor receptor, cathepsin-D, and p53 in saliva among women with breast carcinoma. Cancer Invest..

[91] Brooks M.N., Wang J., Li Y., Zhang R., Elashoff D., Wong D.T. (2008). Salivary protein factors are elevated in breast cancer patients. Mol. Med. Rep..

[92] Wood N., Streckfus C.F. (2015). The Expression of Lung Resistance Protein in Saliva: A Novel Prognostic Indicator Protein for Carcinoma of the Breast. Cancer Invest..

[93] Ohshiro K., Rosenthal D.I., Koomen J.M., Streckfus C.F., Chambers M., Kobayashi R., El-Naggar A.K. (2007). Pre-analytic saliva processing affect proteomic results and biomarker screening of head and neck squamous carcinoma. Int. J. Oncol..

[94] Hu S., Arellano M., Boontheung P., Wang J., Zhou H., Jiang J., Elashoff D., Wei R., Loo J.A., Wong D.T. (2008). Salivary proteomics for oral cancer biomarker discovery. Clin. Cancer Res..

[95] Dowling P., Wormald R., Meleady P., Henry M., Curran A., Clynes M. (2008). Analysis of the saliva proteome from patients with head and neck squamous cell carcinoma reveals differences in abundance levels of proteins associated with tumour progression and metastasis. J. Proteomics.

[96] Rai B., Kaur J., Jacobs R., Anand S.C. (2011). Adenosine deaminase in saliva as a diagnostic marker of squamous cell carcinoma of tongue. Clin. Oral. Investig..

[97] Elashoff D., Zhou H., Reiss J., Wang J., Xiao H., Henson B., Hu S., Arellano M., Sinha U., Le A. (2012). Prevalidation of salivary biomarkers for oral cancer detection. Cancer Epidemiol. Biomarkers Prev..

[98] Winck F.V., Prado Ribeiro A.C., Ramos Domingues R., Ling L.Y., Riano-Pachon D.M., Rivera C., Brandao T.B., Gouvea A.F., Santos-Silva A.R., Coletta R.D. (2015). Insights into immune responses in oral cancer through proteomic analysis of saliva and salivary extracellular vesicles. Sci. Rep..

[99] Wu Z.Z., Wang J.G., Zhang X.L. (2009). Diagnostic model of saliva protein finger print analysis of patients with gastric cancer. World J. Gastroenterol..

[100] Xiao H., Zhang Y., Kim Y., Kim S., Kim J.J., Kim K.M., Yoshizawa J., Fan L.Y., Cao C.X., Wong D.T. (2016). Differential proteomic analysis of Human saliva using tandem mass tags quantification for gastric cancer detection. Sci. Rep..

[101] Xiao H., Zhang L., Zhou H., Lee J.M., Garon E.B., Wong D.T. (2012). Proteomic analysis of human saliva from lung cancer patients using two-dimensional difference gel electrophoresis and mass spectrometry. Mol. Cell Proteomics.

[102] Sun Y., Xia Z., Shang Z., Sun K., Niu X., Qian L., Fan L.Y., Cao C.X., Xiao H. (2016). Facile preparation of salivary extracellular vesicles for cancer proteomics. Sci. Rep..

[103] Chen D.X., Schwartz P.E., Li F.Q. (1990). Saliva and serum CA 125 assays for detecting malignant ovarian tumors. Obstet. Gynecol..

[104] Taylor D.D., Lyons K.S., Gercel-Taylor C. (2002). Shed membrane fragment-associated markers for endometrial and ovarian cancers. Gynecol. Oncol..

[105] Palanisamy V., Sharma S., Deshpande A., Zhou H., Gimzewski J., Wong D.T. (2010). Nanostructural and transcriptomic analyses of human saliva derived exosomes. PLoS ONE.

[106] Sharma S., Rasool H.I., Palanisamy V., Mathisen C., Schmidt M., Wong D.T., Gimzewski J.K. (2010). Structural-mechanical characterization of nanoparticle exosomes in human saliva, using correlative AFM, FESEM, and force spectroscopy. ACS Nano.

[107] Sharma S., Gillespie B.M., Palanisamy V., Gimzewski J.K. (2011). Quantitative nanostructural and single-molecule force spectroscopy biomolecular analysis of human-saliva-derived exosomes. Langmuir.

[108] Han X., Wang J., Sun Y. (2017). Circulating tumor dna as biomarkers for cancer detection. Genomics Proteomics Bioinformatics.

[109] Wang Y., Springer S., Mulvey C.L., Silliman N., Schaefer J., Sausen M., James N., Rettig E.M., Guo T., Pickering C.R. (2015). Detection of somatic mutations and HPV in the saliva and plasma of patients with head and neck squamous cell carcinomas. Sci. Transl. Med..

[110] Basik M., Aguilar-Mahecha A., Rousseau C., Diaz Z., Tejpar S., Spatz A., Greenwood C.M., Batist G. (2013). Biopsies: next-generation biospecimens for tailoring therapy. Nat. Rev. Clin. Oncol..

[111] Bidard F.C., Weigelt B., Reis-Filho J.S. (2013). Going with the flow: from circulating tumor cells to DNA. Sci. Transl. Med..

[112] Pantel K., Alix-Panabieres C. (2013). Real-time liquid biopsy in cancer patients: fact or fiction?. Cancer Res..

[113] Lin M., Song P., Zhou G., Zuo X., Aldalbahi A., Lou X., Shi J., Fan C. (2016). Electrochemical detection of nucleic acids, proteins, small molecules and cells using a DNA-nanostructure-based universal biosensing platform. Nat. Protoc..

[114] Odenthal K.J., Gooding J.J. (2007). An introduction to electrochemical DNA biosensors. Analyst.

[115] Soleymani L., Fang Z., Sargent E.H., Kelley S.O. (2009). Programming the detection limits of biosensors through controlled nanostructuring. Nat. Nanotechnol..

[116] Wei F., Lin C.C., Joon A., Feng Z., Troche G., Lira M.E., Chia D., Mao M., Ho C.L., Su W.C. (2014). Noninvasive saliva-based EGFR gene mutation detection in patients with lung cancer. Am. J. Respir. Crit. Care Med..

[117] Pu D., Liang H., Wei F., Akin D., Feng Z., Yan Q., Li Y., Zhen Y., Xu L., Dong G. (2016). Evaluation of a novel saliva-based epidermal growth factor receptor mutation detection for lung cancer: A pilot study. Thorac Cancer.

[118] Wei F., Yang J., Wong D.T. (2013). Detection of exosomal biomarker by electric field-induced release and measurement (EFIRM). Biosens. Bioelectron..

[119] Inal C., Yilmaz E., Piperdi B., Perez-Soler R., Cheng H. (2015). Emerging treatment for advanced lung cancer with EGFR mutation. Expert Opin. Emerg. Drugs.

[120] Liga A., Vliegenthart A.D., Oosthuyzen W., Dear J.W., Kersaudy-Kerhoas M. (2015). Exosome isolation: A microfluidic road-map. Lab. Chip..

